# Rare *AFG3L2* and *POLG* Variants Suggest a Role for Mitochondrial Dysfunction in Enteric Neuronal Vulnerability in Idiopathic Achalasia

**DOI:** 10.3390/ijms27156756

**Published:** 2026-07-28

**Authors:** Anna Latiano, Francesca Tavano, Lucia Micale, Luigi Bisceglia, Tommaso Biagini, Giulia Mantini, Marco Gentile, Antonio Merla, Fabrizio Bossa, Giuseppe Biscaglia, Tiziana Latiano, Alessandra Pia Bisceglia, Vito Annese, Marco Castori, Tommaso Mazza, Orazio Palmieri

**Affiliations:** 1 Gastrointestinal Disorders Research Unit, Fondazione IRCCS Casa Sollievo della Sofferenza, 71013 San Giovanni Rotondo, Italy; f.tavano@operapadrepio.it (F.T.); tiziana.latiano@gmail.com (T.L.); ap.bisceglia@operapadrepio.it (A.P.B.); o.palmieri@operapadrepio.it (O.P.); 2 Inborn Errors of Morphogenesis Research Unit, Division of Medical Genetics, Fondazione IRCCS Casa Sollievo della Sofferenza, 71013 San Giovanni Rotondo, Italy; l.micale@operapadrepio.it (L.M.); l.bisceglia@operapadrepio.it (L.B.); m.castori@operapadrepio.it (M.C.); 3 Computational Biology and Bioinformatics Unit, Fondazione Policlinico Universitario A. Gemelli IRCCS, 00168 Rome, Italy; tommaso.biagini@policlinicogemelli.it (T.B.); giulia.mantini@policlinicogemelli.it (G.M.); tommaso.mazza@policlinicogemelli.it (T.M.); 4 Division of Gastroenterology and Endoscopy, Fondazione IRCCS Casa Sollievo della Sofferenza, 71013 San Giovanni Rotondo, Italy; m.gentile@operapadrepio.it (M.G.); antoniomerla@hotmail.it (A.M.); f.bossa@operapadrepio.it (F.B.); giuseppe.biscaglia@gmail.com (G.B.); 5 Gastroenterology and Gastrointestinal Endoscopy Unit, IRCCS Policlinico San Donato, 20097 San Donato Milanese, Italy; vito.annese@grupposandonato.it

**Keywords:** achalasia, exome sequencing, in silico analysis, rare variants, mitochondrial dysfunction, inhibitory neurons

## Abstract

Idiopathic achalasia is a rare esophageal motility disorder characterized by the selective degeneration of inhibitory myenteric neurons. Its genetic basis remains poorly defined. We investigated whether rare coding variants may contribute to disease susceptibility. Exome sequencing was performed in 31 individuals with idiopathic achalasia and seven unaffected relatives. Candidate variants were prioritized using phenotype-driven filtering and assessed through in silico and structural analysis of publicly available gene expression and single-cell transcriptomic datasets. No pathogenic or likely pathogenic variants were identified in achalasia-associated genes. A gene-agnostic analysis identified two rare heterozygous missense variants in unrelated patients: *AFG3L2* c.2105G>A (p.Arg702Gln) and *POLG* c.1760C>T (p.Pro587Leu). Both genes encode mitochondrial proteins involved in neuronal homeostasis. The variants affected conserved residues, mapped to functionally relevant protein regions, and were predicted by multiple computational approaches to affect protein stability and function. Both genes were highly expressed in esophageal tissue, with *AFG3L2* showing enrichment in enteric neuronal populations. These exploratory findings support a potential link between mitochondrial dysfunction, enteric neurodegeneration, and idiopathic achalasia. Rare mitochondrial-related variants may contribute to disease susceptibility in selected individuals by increasing vulnerability of inhibitory enteric neurons, although functional validation and larger studies are required. Accordingly, our findings should be considered hypothesis-generating rather than evidence of causality.

## 1. Introduction

Achalasia (AC) is an esophageal motility disorder characterized by absent esophageal body peristalsis and failure of lower esophageal sphincter relaxation, secondary to degeneration of the inhibitory myenteric plexus that innervates the sphincter and the esophageal body [1]. This rare disorder typically presents with dysphagia, weight loss, and regurgitation. AC may be primary if of unknown origin (i.e., idiopathic AC), or secondary when it is interpreted as the consequence of other pre-existing conditions such as cancer and other gastrointestinal or neuromuscular disorders [2].

While the etiology of idiopathic AC remains largely unknown, accumulating evidence points to a multifactorial origin. Familial aggregation and the existence of monogenic syndromes featuring AC support a genetic contribution, at least in selected cases [3,4]. Accordingly, a recent genome-wide association study in 4602 European individuals identified a single nucleotide polymorphism in *HLA-DQB1* and three novel variants outside the HLA locus associated with AC [5]. Despite this, the contribution of inherited variants in idiopathic AC remains largely speculative.

In the present study, we performed clinical exome sequencing in a cohort of 31 individuals with idiopathic AC and available unaffected relatives, with the aim of investigating whether rare coding variants may contribute to disease susceptibility. We identified rare variants in the *AFG3L2* and *POLG* genes in two unrelated patients with AC. Notably, both genes play key roles in mitochondrial homeostasis and maintenance of mitochondrial DNA integrity and have previously been implicated in monogenic neurodegenerative disorders, supporting a potential link between mitochondrial dysfunction and enteric neurodegeneration in AC.

## 2. Results

### 2.1. Molecular Findings

Following variant filtering and annotation using a virtual multigene panel of AC-associated genes derived from the HPO, no likely pathogenic or pathogenic variants were identified according to the ACMG/AMP rules.

A gene-agnostic analysis revealed two rare, deleterious missense changes in two unrelated affected individuals.

One individual (Patient 1) carried the heterozygous c.2105G>A missense variant in exon 16 of the AFG3-like matrix AAA peptidase (*AFG3L2*) gene (NM_006796.3) at position chr18(GRCh37):g.12337410 (Supplementary Figure S1A). The variant leads to an amino-acid substitution from arginine to glutamine at codon 702 (p.Arg702Gln). This variant (rs151344523) is extremely rare according to gnomAD v4.1.0 (2 of 1,179,876 alleles; allele frequency (AF) ≈ 1.7 × 10^−6^; PM2_Supporting) and computational prediction tools supported a deleterious effect (PP3_Supporting). The c.2105G>A variant is reported in ClinVar as likely pathogenic/pathogenic (ID: 5473; 1 star) and has been previously reported as pathogenic in *AFG3L2*-related neurodegenerative disorders (PS4_Strong) [6,7,8,9] (Table 1). Since no established gene–disease relationship currently exists between *AFG3L2* and AC, the significance of this observation requires further studies either supporting a new genotype-phenotype correlation or the hypothesis that AC is an underreported feature of *AFG3L2*-related neurodegenerative disorders.

The patient was a 42-year-old man with a five-year history of dyspeptic symptoms, initially attributed to gastroesophageal reflux disease associated with hiatal hernia and treated with proton-pump inhibitors and prokinetics for three years. He presented to our institution because of worsening symptoms, a sensation of food sticking in the throat, and progressive difficulty swallowing. He had lost 10 kg since the symptoms began to worsen. No suggestive family history was reported. He underwent a thorough evaluation with esophagogastroduodenoscopy, barium swallow, and manometry, which confirmed type I AC according to the Chicago classification. The patient underwent laparoscopic Heller myotomy one year later. In the following years, dysesthesia in the left foot was documented. Ten years later, he presented with hearing loss and tinnitus. Clinical characteristics were prospectively collected by medical history questionnaires.

In a second affected subject (Patient 2), the heterozygous c.1760C>T missense variant in exon 10 of DNA polymerase gamma (*POLG*) gene (NM_002693.3) at position chr15(GRCh37):g.8986887 was identified (Supplementary Figure S1B). This missense change leads to an amino-acid substitution from proline to leucine at codon 587 (p.Pro587Leu). The variant (rs113994096) was rare according to gnomAD v4.1.0 (2884 of 1,179,992 alleles; AF ≈ 2.4 × 10^−3^; PM2_Supporting) and was reported in dbSNP and ClinVar as likely pathogenic/pathogenic (ID: 13505; 1 star). This variant has been reported multiple times as deleterious in the literature following both autosomal dominant and recessive inheritance patterns and was associated with *POLG*-related spectrum disorders [10,11,12]. In silico prediction tools support a deleterious effect (PP3_Supporting). The variant was previously classified as pathogenic/likely pathogenic (PS4_Strong; PM2_Supporting; PP3_Supporting) in mitochondrial or neurological disorders. Future studies are needed to establish whether this finding expands the phenotypic spectrum of *POLG*-related neurodegenerative disorders by identifying a novel genotype–phenotype correlation or indicates that AC is an underrecognized clinical manifestation of these conditions.

The patient was a 46-year-old woman with a two-year history of gradually worsening dysphagia and significant weight loss of approximately 10 kg due to dietary changes with the introduction of a semisolid diet. She presented to our institution where, after evaluation with esophagogastroduodenoscopy, barium swallow, and manometry, she was diagnosed with type I esophageal AC. Thyroid disease was also documented. She subsequently underwent laparoscopic Heller myotomy at the same hospital, where she is currently being treated.

Both patients underwent a neurological examination to exclude the other features characterizing the clinical spectra associated with *POLG* and *AFG3L2*, and were found unaffected. None of the sequenced relatives belonged to the two patients carrying the *AFG3L2* or *POLG* variants; therefore, segregation analysis could not be performed.

### 2.2. In Silico and Structural Characterization of the AFG3L2 (p.Arg702Gln) and POLG (p.Pro587Leu) Variants

Both variants showed an aggregate predictive profile consistent with a deleterious effect, with 16 and 15 predictors indicating a damaging effect for each variant for *AFG3L2* and *POLG*, respectively (Table 2).

The REVEL score was 0.795 for *AFG3L2* (p.Arg702Gln) and 0.796 for *POLG* (p.Pro587Leu), with CADD PHRED values of 33.0 and 29.8, respectively. PolyPhen-2 HDIV reached the maximum score (1.000) for both variants, and PROVEAN was strongly deleterious for both *POLG* (p.Pro587Leu) (−6.69) and *AFG3L2* (p.Arg702Gln) (−3.64). AlphaMissense classified *AFG3L2* (p.Arg702Gln) as likely pathogenic (score 0.651). For *POLG* (p.Pro587Leu), AlphaMissense assigned a score of 0.233 (likely benign class), discordant with the remaining predictors and to be interpreted with caution given the known functional complexity of the *POLG* locus and the experimental biochemical characterization available in the literature for this variant. Both residues showed maximal evolutionary conservation (PhastCons100way = 1.000 for both), with PhyloP100way values of 7.537 (*AFG3L2*) and 9.998 (*POLG*); GERP++ RS values (5.62 and 4.04, respectively) confirmed strong evolutionary constraint.

**Figure 1:**
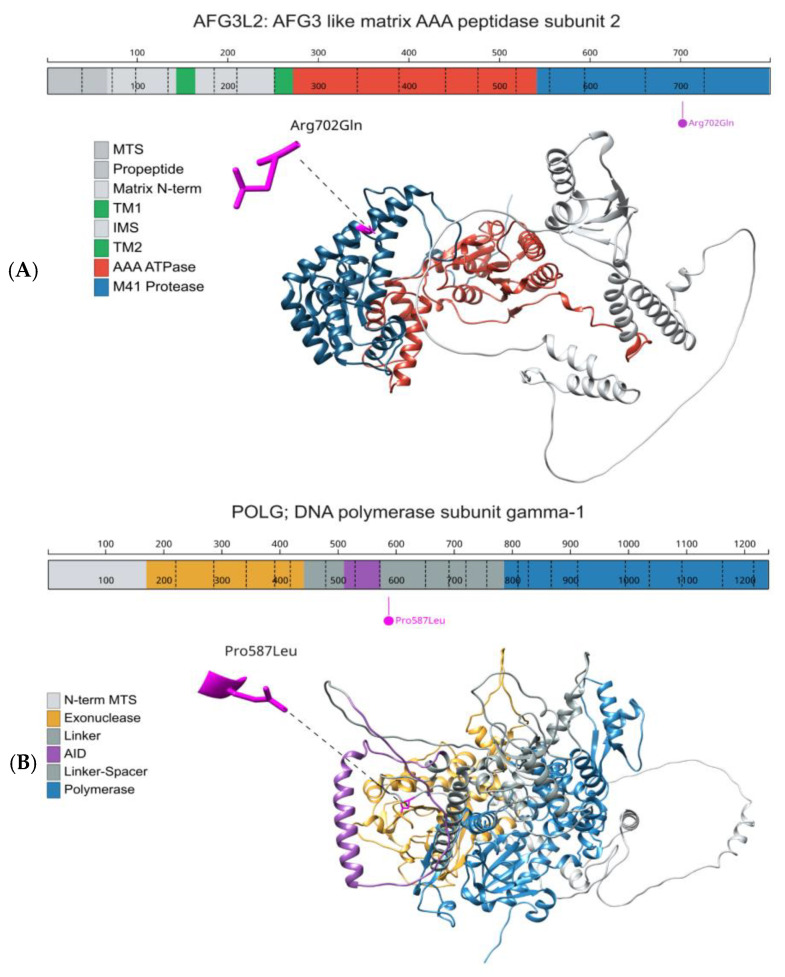
Functional-domain localization and three-dimensional structural mapping of *AFG3L2* (p.Arg702Gln) and *POLG* (p.Pro587Leu). (**A**) Linear domain map of *AFG3L2* (797 aa; NM_006796.3; UniProt Q9Y4W6); the (p.Arg702Gln) variant (magenta) maps to the M41 proteolytic domain. (**B**) Linear domain map of *POLG* (1239 aa; NM_002693.3; UniProt P54098); the (p.Pro587Leu) variant (magenta) maps to the linker/spacer region, adjacent to the RNA-primer (Lys579) and DNA-template (Thr593) binding sites. AlphaFold2 three-dimensional models (EBI) are shown with domain coloring consistent with the linear diagrams and the mutated residue highlighted in magenta. Domain boundaries: UniProt (June 2026). Linear diagrams: ProteinPaint (St. Jude Children’s Research Hospital). Three-dimensional models: UCSF Chimera 1.19. Regions with pLDDT < 70 are not shown.

Functional-domain mapping localized the *AFG3L2* (p.Arg702Gln) variant within the M41 proteolytic domain (residues 541–797), in the α-helix spanning residues 691–718 (Figure 1A), whereas the *POLG* (p.Pro587Leu) variant mapped to the linker/spacer region, between the accessory-interacting determinant (AID, 510–571) and the polymerase domain (786–1239), adjacent to the RNA-primer (Lys579) and DNA-template (Thr593) binding sites (Figure 1B). The AlphaFold2 models showed high pLDDT scores (≥90) in the regions surrounding both mutated residues, confirming that positions 702 and 587 lie within well-defined structural domains with high predictive confidence.

FoldX stability analysis predicted a ΔΔG of +1.198 ± 0.048 kcal/mol for *AFG3L2* (p.Arg702Gln), classified as destabilizing (|ΔΔG| > 1.0 kcal/mol); the Arg → Gln substitution at residue 702, located in the long α-helix of the M41 domain, is predicted to compromise the thermodynamic stability of the mitochondrial AAA protease. For *POLG* (p.Pro587Leu) the ΔΔG was +0.767 ± 0.005 kcal/mol (mildly destabilizing); the loss of conformational rigidity conferred by the proline residue in the linker region is consistent with a local alteration that may impair functional coordination between the exonuclease and polymerase domains. DynaMut2 concordantly predicted a destabilizing direction for both variants (ΔΔG = −0.69 kcal/mol for *AFG3L2*, destabilizing; −0.08 kcal/mol for *POLG*, marginally destabilizing) (Table 3).

Missense3D did not identify significant local structural perturbations for either variant (neutral in both cases).

The three approaches probe distinct, non-redundant aspects of the structural impact: Missense 3D evaluates discrete categories of local perturbation, whereas FoldX and DynaMut2 quantify the global change in folding free energy. Taken together and in light of this, the structural evidence is concordant and moderately destabilizing for *AFG3L2* (p.Arg702Gln), where two independent thermodynamic predictors (FoldX +1.198 ± 0.048 kcal/mol; DynaMut2 −0.69 kcal/mol) agree on a destabilizing direction in the absence of an overt local defect, consistent with a subtle but reproducible impact on the M41 protease α-helix. For *POLG* (p.Pro587Leu) the picture is weaker: FoldX predicts a mildly destabilizing effect (+0.767 ± 0.005 kcal/mol), DynaMut2 a marginal one (−0.08 kcal/mol), and Missense3D detects no local perturbation, a pattern consistent with a modest, possibly context-dependent effect on protein structure rather than a major destabilization.

### 2.3. Tissue-Specific Expression Profiling of POLG and AFG3L2

**Figure 2:**
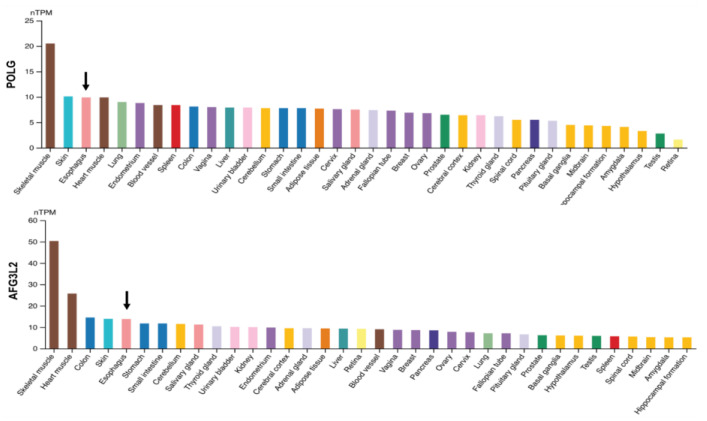
Baseline tissue expression of *POLG* and *AFG3L2* across healthy human tissues. Bar charts of normalized RNA expression (nTPM) for *POLG* (**top**) and *AFG3L2* (**bottom**) ranked across human tissues. Expression values were obtained from the Human Protein Atlas (HPA) consensus dataset, which integrates RNA-seq data from GTEx, HPA, and FANTOM5 using an internal normalization pipeline. Arrows indicate the esophagus, which ranks third *(POLG)* and fifth *(AFG3L2)* among all tissues.

To investigate whether public evidence linking the candidate variants to gene expression was available, we searched the GTEx portal for corresponding eQTL data. However, owing to the extreme rarity of these variants, no direct eQTL information was available. Thus, no inference regarding the effects of these variants on gene expression could be made. Therefore, we assessed the physiological baseline expression of the candidate genes across healthy human tissues (Figure 2). This analysis revealed a distinct tissue distribution: *POLG* and *AFG3L2* expression in the esophagus ranked among the most highly expressed across the body (third for *POLG* and fifth for *AFG3L2*), suggesting a likely physiological role within the upper gastrointestinal tract.

### 2.4. Cell-Type-Specific Enrichment of AFG3L2 in the Enteric Nervous System

**Figure 3:**
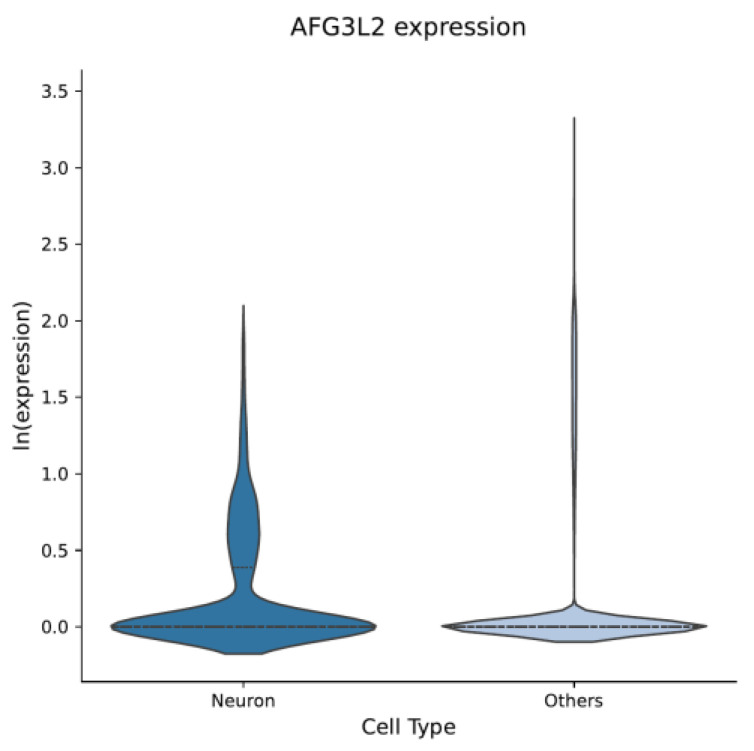
Cell-type-specific expression of *AFG3L2* in the human enteric nervous system. *AFG3L2* expression in neurons versus all other cell types in a single-cell RNA-sequencing dataset of the human enteric nervous system (dataset id: SCP1038, the Single Cell Portal).

To further define the cellular localization of the candidate genes within the gut microenvironment, we analyzed a single-cell RNA-sequencing dataset of the human enteric nervous system [13]. This analysis showed that *AFG3L2* is more highly expressed in neurons compared with all other cell types (Figure 3). These results support a preferential expression of *AFG3L2* in enteric neurons, suggesting a specific role in neuronal mitochondrial function within the ENS. The same consideration does not hold for *POLG*, which showed no detectable expression across all cell types in this dataset.

## 3. Discussion

Idiopathic AC is considered a disorder of the inhibitory and excitatory neurotransmitters in the distal esophagus, secondary to loss of ganglion cells. The underlying etiology may be attributed to an abnormal inflammatory response, autoimmunity, or an infectious trigger [14]. In the absence of these acquired factors, reports of familial recurrence support a genetic background. Accordingly, AC is occasionally part of other complex conditions or multisystem disorders characterized by generalized autonomic or neuromuscular dysfunction (e.g., familial dysautonomia, glucocorticoid insufficiency, Allgrove syndrome, Down syndrome) [15]. In recent years, the enteric nervous system has been consistently reported to play a coordinating and controlling role in gastrointestinal motility. Motility disorders are closely related to functional or anatomical changes in the gastrointestinal nervous system, and gastrointestinal motility disorders are well documented in neurological diseases such as stroke, parkinsonism, multiple sclerosis, and diabetic neuropathy [16].

Mitochondrial dysfunction is a key factor in the pathophysiology of multiple neurodegenerative diseases. Alterations in mitochondrial dynamics and axonal transport, as well as mitochondrial DNA (mtDNA) variants, have been associated with impaired mitochondrial function [17]. Mitochondrial disorders (MIDs) can be caused by genetic defects in mtDNA, small nucleotide variants, deletions, and mtDNA depletions, with autosomal dominant, autosomal recessive, or X-linked inheritance patterns [18], or in nuclear genes encoding proteins responsible for the maintenance of integrity and biogenesis of mtDNA. MIDs frequently display marked tissue specificity and variable penetrance despite broad expression of disease-associated genes. This phenotypic heterogeneity likely reflects differences in cellular energetic requirements, mitochondrial dependence, tissue-specific compensatory mechanisms, and additional genetic or environmental modifiers [17,19]. The tissues most commonly affected by mtDNA depletion syndromes and related disorders include the central and peripheral nervous system, eyes, ears, liver, muscles, and intestinal tract. Gastrointestinal manifestations are common in MIDs and include poor appetite, gastroesophageal sphincter dysfunction, constipation, dysphagia, vomiting, gastroparesis, GI pseudo-obstruction, diarrhea, pancreatitis, and hepatopathy [20]. AC has been only occasionally reported in both pediatric and adult MIDs, always within pleiotropic presentations. The presence of gastrointestinal dysmotility, peripheral neuropathy, and generalized autonomic dysfunction led to the diagnosis of a MID in two siblings with AC manifesting in early childhood [21], while cricopharyngeal AC has been reported in Kearns–Sayre syndrome (OMIM 530000) and progressive external ophthalmoplegia (OMIM 609286) due to single large-scale mtDNA deletions [22].

This report describes, for the first time, the occurrence of previously characterized rare variants, associated with *AFG3L2*-related autosomal dominant spinocerebellar ataxia and *POLG*-spectrum disorders, in two unrelated individuals with apparently isolated idiopathic AC. Rather than representing monogenic causes of AC, the *AFG3L2* and *POLG* variants identified in this study are more plausibly interpreted as low-frequency genetic susceptibility factors that may increase the vulnerability of enteric neurons through subtle impairment of mitochondrial homeostasis. This hypothesis is consistent with the emerging view of idiopathic AC as a complex multifactorial disorder, in which genetic predisposition interacts with immune-mediated mechanisms and environmental triggers. Indeed, the recent genome-wide association study by Grover et al. [5] identified multiple susceptibility loci and polygenic risk, predominantly involving immune-related pathways, supporting a polygenic architecture underlying disease risk. Within this framework, rare mitochondrial-related variants such as those described here, may represent one component of a broader pathogenic network, acting in concert with common susceptibility alleles and inflammatory processes to reduce neuronal resilience and promote selective degeneration of inhibitory myenteric neurons. Although this model remains speculative, it provides a biologically plausible mechanism linking mitochondrial dysfunction to the selective neuronal vulnerability that characterizes AC and offers a conceptual framework for future genetic and functional studies. More generally, our findings raise the possibility that idiopathic AC may represent a previously understudied manifestation within the phenotypic spectrum associated with deleterious variants in *AFG3L2* and *POLG*, potentially linking mitochondrial metabolic impairment to enteric neurodegeneration, and may even represent an early, isolated, or prodromal manifestation preceding the development of overt neurodegenerative disorders or MIDs.

*AFG3L2* is located on chromosome 18p11.21 and encodes a mitochondrial ATPase belonging to the AAA protease family localized in the inner mitochondrial membrane. It acts as an ATP-dependent protein quality-control system, processes membrane-inserted protein substrates, and is essential for axonal and neuronal development [23]. Deleterious variants in *AFG3L2* are linked to several neurodegenerative disorders, including autosomal dominant optic atrophy (OMIM 618977), autosomal dominant spinocerebellar ataxia (SCA28, OMIM 610246) [6], and autosomal recessive spastic ataxia (SPAX5, OMIM 614487). Although there are currently no documented cases linking abnormal *AFG3L2* genotypes with AC, mild dysphagia has occasionally been reported in *AFG3L2*-related conditions [24,25,26]. While it cannot be established whether the dysphagia reported in previously described *AFG3L2*-related disorders resulted from impaired inhibitory–excitatory neurotransmission secondary to enteric ganglion-cell loss in the distal esophagus, our observation, together with the predicted destabilization of the M41 protease domain (Figure 1A) and the marked enrichment of *AFG3L2* in enteric neurons (Figure 3), raises the possibility that AC may, in some cases, represent a prominent clinical manifestation in adults harboring rare heterozygous *AFG3L2* variants.

*POLG* maps to chromosome 15q26.1, encodes the catalytic subunit of DNA polymerase gamma, and is involved in the replication and repair of mtDNA [27]. Most alterations described in *POLG* affect the functional exonuclease and polymerase domains and are associated with marked clinical heterogeneity [28]. Deleterious variants in *POLG* are associated with autosomal dominant and recessive progressive external ophthalmoplegia (OMIM 157640, OMIM 258450), mitochondrial recessive ataxia syndrome (OMIM 607459), and mtDNA depletion syndromes 4A (OMIM 203700) and 4B (OMIM 613662), collectively termed *POLG*-related disorders [11,19]. While speech and swallowing difficulties are common in *POLG*-related disorders such as mitochondrial recessive ataxia syndrome [29], AC has been rarely described; two studies reported patients presenting with AC and with typical features of MIDs and heterogeneous phenotypes, carrying *POLG* variants [30,31]. Our patient further supports the observation that AC may occasionally represent a presenting manifestation in adults carrying heterozygous *POLG*-related mitochondrial variants. The (p.Pro587Leu) substitution maps to the linker/spacer region adjacent to the substrate-binding sites and is predicted to mildly destabilize the protein (Figure 1B). However, the relatively high population frequency of the *POLG* allele argues against a fully penetrant monogenic role in AC and instead supports the hypothesis that this variant may act as a low-penetrance susceptibility or risk-modifying mitochondrial allele, potentially contributing to disease in combination with additional genetic, epigenetic, or environmental factors.

With this report, we suggest a potential genetic and pathogenetic link between idiopathic AC, neurodegenerative disorders and mitochondrial dysfunction. A hypothetical conceptual bridge is that variants in *AFG3L2* and *POLG* may impair mitochondrial quality control, mtDNA maintenance, and neuronal bioenergetics, ultimately contributing to degeneration of inhibitory myenteric neurons, esophageal dysmotility, failure of lower esophageal sphincter relaxation, and loss of peristalsis, thus contributing to AC and other motility disorders [18,32]. Consistent with a tissue- and cell-context-dependent effect, both genes are highly expressed in esophageal tissue (Figure 2) and *AFG3L2* is enriched in enteric neurons (Figure 3). Given that *AFG3L2* variants have been shown to be detrimental in specific cellular contexts such as Purkinje neurons [6], other substrates that differ in origin, function, and metabolic requirements, such as inhibitory neurons of the esophageal myenteric plexus, could be tested. Enteric inhibitory neurons, particularly nitrergic NOS1+ neurons, may represent a metabolically vulnerable population because of their high energetic demands, dependence on oxidative phosphorylation, calcium-buffering requirements, and constitutive nitric-oxide signaling, all of which increase susceptibility to mitochondrial defects and oxidative stress. Selective vulnerability of these populations has been increasingly implicated in enteric neuropathies and gastrointestinal motility disorders [33,34]. In this context, mitochondrial dysfunction could promote impaired neurotransmission, defective neuronal maintenance, and progressive neuronal loss within the esophageal myenteric plexus, thereby contributing to failure of lower esophageal sphincter relaxation and loss of peristalsis in AC.

Several limitations should be acknowledged in this study. The relatively limited sample size reduced statistical power and did not allow extensive testing of the frequency of rare variants. Segregation studies could not be conducted due to the lack of DNA from relatives of all affected individuals and their limited clinical evaluation. We acknowledge that the absence of clinical abnormalities and the lack of extensive neuromuscular and biochemical investigations cannot exclude subtle or age-dependent manifestations, particularly considering the incomplete penetrance and variable expressivity of *AFG3L2*- and *POLG*-related disorders. Importantly, the structural, conservation, and expression analyses presented here are computational and hypothesis-generating. In silico analyses provide evidence supporting the functional impact of the two variants, but they cannot replace functional validation. Functional studies, including assessments of mitochondrial respiration, mtDNA stability, neuronal viability, and enteric neuronal function, are still lacking, so the pathogenic relevance of these variants in esophageal myenteric neurons remains hypothetical. Exome sequencing also does not comprehensively evaluate mtDNA variants, structural variants, repeat expansions, epigenetic alterations, or non-coding regulatory regions. Finally, the absence of replication in an independent cohort limits generalizability and requires cautious interpretation.

## 4. Materials and Methods

### 4.1. Sample Collection

Exome sequencing was carried out on 31 unrelated patients with AC and seven unaffected relatives including 1 mother and 6 sisters, each related to a different affected individual. The unaffected relatives were included to facilitate family-based interpretation of candidate variants and to evaluate segregation whenever informative family structures were available. Patient characteristics are summarized in Table 4.

Patients with idiopathic AC were diagnosed using high-resolution esophageal manometry, supported by clinical history and barium esophagogram. To exclude secondary forms of AC, esophagogastroscopy and/or computed tomography or magnetic resonance imaging were performed when required. Further clinical characteristics were prospectively collected using standardized medical history questionnaires. Peripheral blood samples were collected from patients and healthy relatives for DNA extraction and stored at −80 °C until analysis.

### 4.2. Exome Sequencing

Genomic DNA was extracted from peripheral blood samples using the QIAamp DNA Blood Kit (Qiagen, Hilden, Germany) according to the manufacturer’s instructions. DNA concentration and purity were assessed spectrophotometrically using a NanoDrop 2000C spectrophotometer (Thermo Fisher Scientific, Waltham, MA, USA). Libraries were prepared using the Agilent Bravo semi-automated platform and the SureSelect Custom Constitutional Panel 17 Mb (CCP17) (Agilent Technologies, Santa Clara, CA, USA), a clinical exome assay targeting more than 5000 disease-associated genes. Sequencing was performed on an Illumina NextSeq 500 platform (Illumina, San Diego, CA, USA) using Mid Output v2.5 flow cells (300 cycles), achieving a mean target coverage depth of ≥100×. Run quality metrics showed an overall Q30 score of 91.37%, an average sequencing error rate of 0.58%, and approximately 91% passing-filter clusters, consistent with a high-quality sequencing run. Raw sequencing data were automatically demultiplexed into FASTQ files and processed using a custom bioinformatics pipeline implemented in Partek Flow v12.11.0 (Partek Incorporated, Chesterfield, MO, USA). Reads underwent quality control, adapter trimming, alignment to the GRCh37/hg19 human reference genome using the Burrows–Wheeler Aligner v0.7.19 (BWA), duplicate read removal, and variant calling using Genome Analysis Toolkit v4.6.2.0 (GATK) HaplotypeCaller. The resulting Variant Call Format (VCF) files were subsequently imported into Agilent Alissa Interpret v5.4.2 for variant annotation, filtering, prioritization, and clinical interpretation. Variant quality filters included variant allele fraction (VAF) ≥ 5%, total read depth ≥ 20×, alternative allele depth ≥10 reads, genotype quality (GQ) ≥ 20, and mapping quality (MQ) ≥ 40.

Variant prioritization was performed in two sequential steps. First, variants were filtered according to a rare Mendelian disease model using minor allele frequency (MAF) thresholds of <0.001 for autosomal dominant and X-linked genes and <0.01 for autosomal recessive genes, based on gnomAD v2.1.1. These allele-frequency thresholds were used as prioritization criteria rather than absolute exclusion criteria. Accordingly, a subsequent gene-agnostic analysis was undertaken to identify variants in genes with established roles in mitochondrial biology and neurological disorders that had previously been reported as pathogenic or likely pathogenic in ClinVar (https://www.ncbi.nlm.nih.gov/clinvar, accessed on 1 January 2026) and/or supported by published functional evidence, irrespective of whether they fulfilled the initial allele-frequency thresholds. For the final annotation of retained variants, allele frequencies were updated using the most recent publicly available databases (including gnomAD v4.1) after verification of transcript and HGVS nomenclature consistency.

Variants were further prioritized if they fulfilled one or more of the following criteria: (i) de novo occurrence in autosomal dominant or X-linked genes, or homozygous/compound heterozygous occurrence in autosomal recessive genes; (ii) previous classification as pathogenic or likely pathogenic in ClinVar or the Leiden Open Variation Database (LOVD; https://www.lovd.nl, accessed on 1 January 2026); (iii) predicted loss-of-function variants (nonsense or frameshift) in genes with established loss-of-function disease mechanisms; (iv) canonical splice-site variants affecting the ±1 or ±2 positions; (v) small in-frame insertions or deletions; or (vi) missense variants predicted to be deleterious by in silico tools (REVEL ≥ 0.644, CADD v1.6 ≥ 25.3 and/or PolyPhen-2 HumVar ≥ 0.978). Intronic, untranslated region, and synonymous variants were excluded unless previously reported as pathogenic.

A phenotype-driven virtual gene panel was generated using Human Phenotype Ontology (HPO) terms including Achalasia (HP:0002571), Dysphagia (HP:0002015), and Feeding difficulties (HP:0011968). Candidate variants emerging from the phenotype-driven analysis and the subsequent gene-agnostic analysis were interpreted according to the American College of Medical Genetics and Genomics/Association for Molecular Pathology (ACMG/AMP) guidelines [35]. The final prioritization integrated population frequency, predicted functional impact, clinical annotations, published disease associations, and biological plausibility in relation to the current understanding of idiopathic AC.

DNA fragments containing the variants to be confirmed were amplified by PCR using primers designed with the Primer3 tool (https://primer3.ut.ee/, (accessed on 1 March 2026)) (*AFG3L2*, NM_006796.3, F: 5′-ATCTCCTTTGACCTCCCACG-3′, R: 5′-AACTGGCACCTACCTTCTCC-3′; *POLG*, NM_002693.3, F: 5′-CGTAGATGGTACCGGAAGCT-3′, R: 5′-GCCATGACGCTCTGAGTAGT-3′). The PCRs were performed on a GeneAmp^®^ PCR Systems 9700 (Applied Biosystems, Foster City, CA, USA) in a final volume of 25 µL containing 10× Buffer, 15 mM dNTP, 25 mM MgCl_2_, 10 ng/µL genomic DNA, 5 U/µL AmpliTaq Gold DNA, and 15 pmol primers F and R. Conditions were 94 °C for 10 min followed by 35 cycles at 94 °C for 1 min, 62 °C for 1 min, 72 °C for 1 min and a final step at 72 °C for 10 min. PCR products (172 bp *AFG3L2* and 150 bp *POLG*) were analyzed on a 2% agarose gel to verify size. The PCR products were sequenced on an ABI 3500xL DNA Analyzer (Applied Biosystems, Foster City, CA, USA). Reactions were performed in a final volume of 20 µL containing Big Dye v1.1, 4 µL PCR product, 3.2 pmol primers F and R. Conditions were 96 °C for 1 min followed by 25 cycles at 96 °C for 10 s, 50 °C for 5 s, 60 °C for 4 min. Sequence data were analyzed with Sequencing Analysis software v.5.4.

### 4.3. In Silico Pathogenicity Assessment and Structural Characterization of Candidate Variants

To further characterize the two missense variants identified by exome sequencing, *AFG3L2* NM_006796.3:c.2105G>A (p.Arg702Gln) and *POLG* NM_002693.3:c.1760C>T (p.Pro587Leu), and to address their potential pathogenicity, an integrated in silico analysis was performed. This analytical workflow included pathogenicity prediction using multiple tools, evolutionary-conservation scoring, functional-domain mapping, and three-dimensional structural characterization.

#### 4.3.1. Pathogenicity Prediction

The functional impact of each variant was assessed using a panel of bioinformatic sequence-based and Ensemble/meta-score predictors (Table 2). Each predictor was classified as deleterious or tolerated according to published thresholds, and the total number of concordant deleterious predictions was reported as an aggregate score. Functional classification was additionally obtained using AlphaMissense (https://alphamissense.hegelab.org, accessed on 1 January 2026), a deep-learning model trained on AlphaFold2 representations, which assigns a continuous score (0–1) and one of three classes: likely benign (<0.340), ambiguous (0.340–0.564), or likely pathogenic (>0.564).

#### 4.3.2. Evolutionary Conservation

Evolutionary conservation of each mutated residue was assessed using PhastCons (100-vertebrate and 17-primate alignments), PhyloP (100 vertebrates and 17 primates), GERP++ RS, and bStatistic. PhastCons scores approaching 1 and positive, high PhyloP and GERP++ RS values indicate strong purifying selection at the residue.

#### 4.3.3. Functional Domain Mapping and Annotation

The position of each mutated residue relative to protein functional domains and catalytic sites was determined based on UniProt annotations (accessions Q9Y4W6 for *AFG3L2* and P54098 for *POLG*; June 2026). Domain boundaries were extracted from the UniProt feature tables (Transmembrane, Topological domain, Region, Domain, Active site, and Binding site features). For *AFG3L2* (797 aa), annotated features included the mitochondrial targeting sequence (MTS, residues 1–38), propeptide (39–66), matrix N-terminal region (67–142), transmembrane helix 1 (TM1, 143–163), intermembrane-space domain (IMS, 164–250), transmembrane helix 2 (TM2, 251–271), AAA+ ATPase module (272–540), and M41 proteolytic domain (541–797), with Glu408 (Walker B motif) and Glu575 (HExGH zinc-binding motif) as catalytic reference sites. For *POLG* (1239 aa), annotated features included the N-terminal/MTS region (1–169), 3′→5′ exonuclease domain (170–440; motifs Exo I–III), linker region (441–509), accessory-interacting determinant (AID, 510–571), linker/spacer region (572–785), and polymerase domain (786–1239; motifs Pol A–C), with Asp198 (exonuclease active site) and Asp890/Asp1135 (polymerase active site) as catalytic reference sites. Linear domain diagrams were generated with ProteinPaint v 2.199.0 (St. Jude Children’s Research Hospital), using the curated domain boundaries as custom annotations and mapping each variant as a lollipop marker at the corresponding residue. Domain color assignments were kept consistent between the linear diagrams and the three-dimensional models (Figure 1).

#### 4.3.4. Three-Dimensional Structural Modelling

Three-dimensional models of *AFG3L2* and *POLG* were retrieved from the AlphaFold Protein Structure Database (https://alphafold.ebi.ac.uk, accessed on 1 May 2026; UniProt accessions Q9Y4W6 and P54098) and visualized with UCSF Chimera v1.19. Functional domains were colored according to the UniProt boundaries, consistently with the linear diagrams, and the mutated residue was highlighted in magenta. Model reliability was assessed per residue using the pLDDT (predicted local distance difference test) score: ≥90 very high confidence, 70–90 high, 50–70 low, <50 very low. Regions with pLDDT < 70 were not displayed.

#### 4.3.5. Protein Stability Prediction

The effect of each substitution on protein thermodynamic stability was quantified using two independent tools applied to the AlphaFold2 model. FoldX v5.0 was used to calculate the free-energy change of folding (ΔΔG, kcal/mol) between wild-type and mutant, classified as neutral (|ΔΔG| ≤ 0.6 kcal/mol), mildly destabilizing (0.6 < |ΔΔG| ≤ 1.0 kcal/mol), or destabilizing (|ΔΔG| > 1.0 kcal/mol). DynaMut2, which integrates normal-mode analysis with ΔΔG calculation, was applied in parallel using the same model. FoldX and DynaMut2 adopt opposite sign conventions: a positive ΔΔG in FoldX and a negative ΔΔG in DynaMut2 both indicate a destabilizing effect.

#### 4.3.6. Local Structural Perturbation Analysis

Local structural perturbations induced by each substitution were analyzed using Missense3D v2.0, which systematically evaluates 16 structural criteria on the three-dimensional model, including steric clashes, secondary-structure alterations, introduction of charged or hydrophilic residues in buried regions, disruption of buried hydrogen bonds or salt bridges, disallowed Ramachandran phi/psi angles, and substitution of cis-proline. The overall assessment is reported as Damaging or Neutral.

### 4.4. Tissue-Specific and Single-Cell Gene Expression Analysis

#### 4.4.1. GTEx Query and Tissue-Specific Gene Expression Analysis

Publicly available eQTL information was queried in the GTEx (Genotype-Tissue Expression; www.gtexportal.org, accessed on 1 May 2026) database to determine whether expression quantitative trait locus data were available for the selected variants. Because of the rarity of the identified variants, no direct eQTL information was available. Therefore, no conclusions regarding variant-associated changes in gene expression could be drawn from GTEx data.

#### 4.4.2. Baseline Tissue Expression Profiling of *POLG* and *AFG3L2*

To determine the physiological expression patterns of *POLG* and *AFG3L2* in healthy human tissues, baseline bulk RNA expression data (normalized transcripts per million, nTPM) were retrieved from the Human Protein Atlas consensus dataset (https://www.proteinatlas.org; accessed on 1 May 2026), which integrates RNA-sequencing data from the Human Protein Atlas, GTEx, and FANTOM5 following internal normalization. Gene expression levels were compared across all available human organs and tissue types to identify anatomical sites with relatively high transcript abundance.

#### 4.4.3. Single-Cell RNA Sequencing (scRNA-Seq)

To investigate the cell-type-specific expression profile of *AFG3L2* within the human enteric nervous system (ENS) [13], a publicly available scRNA-seq dataset was downloaded from the Single Cell Portal (https://singlecell.broadinstitute.org/single_cell, accessed on 1 May 2026), dataset id: SCP1038) and analyzed. The dataset comprises 102 biological samples and 146,442 single cells in which *AFG3L2* expression was detected. Quality control and initial preprocessing were performed using an internal Single Cell Portal pipeline. Gene expression values were normalized using a count-per-10,000 (CP10K) scaling followed by log1p transformation. Expression profiles of *AFG3L2* were compared between neuronal and all other combined cell types. The Mann–Whitney U test was applied for descriptive purposes only to support data visualization in violin plots, and not as a formal inferential test for differential expression, as individual cells do not represent independent biological replicates.

## 5. Conclusions

In conclusion, in this exploratory exome-sequencing study of individuals with idiopathic AC, we identified rare heterozygous variants in the mitochondrial-related genes *AFG3L2* and *POLG* in two unrelated patients and supported their plausibility with integrated in silico, structural, and expression analyses. Although these findings do not establish causality, they should be considered as hypothesis-generating, indicating that mitochondrial dysfunction and impaired neuronal bioenergetics may contribute to enteric neurodegeneration in a subset of patients with AC. Since no established gene–disease relationship currently exists between *AFG3L2* or *POLG* and AC, the identified variants should presently be regarded as variants of uncertain significance with respect to the AC phenotype. Larger genetic studies, functional validation, and integration with transcriptomic and cellular analyses will be necessary to clarify the role of mitochondrial pathways in esophageal motility disorders. Future studies should also include a systematic neurological, electrophysiological, and biochemical characterization of individuals carrying candidate variants and a well-documented family history.

## Figures and Tables

**Table 1:** Heterozygous missense variants identified after filtering exome-sequencing data.

Parameter	*AFG3L2*	*POLG*
Chromosomal location (GRCh37)	chr18:12337410	chr15:8986887
cDNA change	NM_006796.3:c.2105G>A	NM_002693.3:c.1760C>T
dbSNP ID	rs151344523	rs113994096
Amino-acid change	p.Arg702Gln	p.Pro587Leu
Exonic function	missense	missense
Inheritance model	AD/AR	AD/AR
gnomAD exomes AF (v4.1.0)	1.7 × 10^−6^	2.4 × 10^−3^
ClinVar classification	Pathogenic/Likely pathogenic	Pathogenic/Likely pathogenic
ACMG classification	Pathogenic (PM2_Supporting, PP3_Supporting, PS4_Strong)	Likely Pathogenic (PM2_Supporting, PP3_Supporting, PS4_Strong)
LOVD classification	Pathogenic	Pathogenic
REVEL score	0.795	0.796
CADD score	33.0	29.8

GRCh37/hg19 used as reference genome. AD: autosomal dominant; AR: autosomal recessive; AF: allele frequency; gnomAD: Genome Aggregation Database; ACMG: American College of Medical Genetics; LOVD: Leiden Open Variation Database; REVEL: Rare Exome Variant Ensemble Learner; CADD: Combined Annotation-Dependent Depletion. Both variants should currently be considered variants of uncertain significance with respect to idiopathic achalasia phenotype, as no disease-specific pathogenicity framework currently exists.

**Table 2:** Integrated in silico pathogenicity analysis of *AFG3L2* (p.Arg702Gln) and *POLG* (p.Pro587Leu).

Parameter	*AFG3L2* (p.Arg702Gln)	*POLG* (p.Pro587Leu)
General information		
Transcript	NM_006796.3	NM_002693.3
cDNA change	c.2105G>A	c.1760C>T
Exon	16/17	10/23
gnomAD v4.1 AF	1.69 × 10^−6^ (2/1,179,876)	2.44 × 10^−3^ (2884/1,179,992)
ClinVar ID (classification)	5473 (Pathogenic/LP)	13505 (Pathogenic/LP)
Evolutionary conservation		
PhastCons100way (vertebrates)	1.000	1.000
PhastCons17way (primates)	0.979	0.949
PhyloP100way (vertebrates)	7.537	9.998
PhyloP17way (primates)	2.910	4.821
GERP++ RS	5.62	4.04
bStatistic	696	940
Sequence-based predictors		
SIFT	0.052 (T)	0.003 (D)
SIFT4G	0.019 (D)	0.003 (D)
PolyPhen-2 HDIV	1.000 (D)	1.000 (D)
PROVEAN	−3.64 (D)	−6.69 (D)
MutationTaster	1.000 (D)	1.000 (D)
MutationAssessor	3.145 (D)	2.810 (D)
Ensemble/meta-score predictors		
REVEL	0.795 (D)	0.796 (D)
CADD PHRED	33.0 (D)	29.8 (D)
BayesDel addAF	0.403 (D)	0.075 (D)
DANN	0.9996 (D)	0.9984 (D)
MetaLR	0.796 (D)	0.912 (D)
VEST4	0.841 (D)	0.812 (D)
ClinPred	0.978 (D)	0.071 (T)
Eigen-PC	1.254 (D)	0.987 (D)
DEOGEN2	0.821 (D)	0.634 (D)
M-CAP	0.261 (D)	0.178 (D)
Structure-based/deep-learning predictor		
AlphaMissense score	0.651	0.233
AlphaMissense class	likely pathogenic	likely benign †

D = damaging; T = tolerated; LP = likely pathogenic; AF = allele frequency. † The AlphaMissense score of 0.233 for *POLG* (p.Pro587Leu) is discordant with the remaining deleterious predictors; the variant is classified as pathogenic in the context of *POLG*-related disorders (ClinVar ID 13505).

**Table 3:** Structural stability analysis of *AFG3L2* (p.Arg702Gln) and *POLG* (p.Pro587Leu).

Parameter	*AFG3L2* (p.Arg702Gln)	*POLG* (p.Pro587Leu)
AlphaFold2—structural-model quality		
Domain localization	M41 protease (α-helix 691–718)	Linker/spacer (β-strand 587–589)
pLDDT at mutated residue	≥90 (very high)	≥90 (very high)
FoldX—ΔΔG > 0 = destabilizing		
ΔΔG (kcal/mol)	+1.198 ± 0.048	+0.767 ± 0.005
Classification	Destabilizing (|ΔΔG| > 1.0)	Mildly destabilizing (0.6–1.0)
DynaMut2—ΔΔG < 0 = destabilizing		
ΔΔG (kcal/mol)	−0.69	−0.08
Classification	Destabilizing	Marginally destabilizing ‡
Missense3D—local structural perturbations		
Result	Neutral	Neutral
Criteria evaluated	16 structural criteria	16 structural criteria

FoldX: ΔΔG > 0 = destabilizing (neutral ≤ 0.6; mildly destabilizing 0.6–1.0; destabilizing > 1.0 kcal/mol). DynaMut2: ΔΔG < 0 = destabilizing (opposite sign convention to FoldX). ‡ The DynaMut2 ΔΔG of −0.08 kcal/mol for *POLG* (p.Pro587Leu) indicates a marginal destabilizing effect; FoldX predicts a mildly destabilizing effect (+0.767 kcal/mol). Both tools concordantly predict a destabilizing direction.

**Table 4:** General characteristics of the 31 patients with achalasia.

Characteristic	Value
Male/female	16/15 (51.6% M)
Mean age, years	63 ± 13.1
Males	65 ± 12
Females	60 ± 14.2
Age at diagnosis, years	47 ± 13.5
Duration of symptoms before diagnosis, years	
≤1	15
>1	16
Dysphagia, *n* (%)	31 (100%)
Esophageal regurgitation, *n* (%)	20 (64.5%)
Chest pain, *n* (%)	3 (9.7%)
Weight loss	
≥5 kg, *n* (%)	21 (67.7%)
<5 kg, *n* (%)	10 (32.2%)
Heller myotomy, *n* (%)	23 (74.2%)
Autoimmune diseases, *n* (%)	5 (16%)

Values expressed as mean ± SD (standard deviation).

## Data Availability

The original contributions presented in this study are included in the article. Further inquiries can be directed to the corresponding author.

## Supplementary Materials

The following supporting information can be downloaded at: https://www.mdpi.com/article/10.3390/ijms27156756/s1.

## References

[1] Boeckxstaens G.E., Zaninotto G., Richter J.E. (2014). Achalasia. Lancet.

[2] Schizas D., Theochari N.A., Katsaros I., Mylonas K.S., Triantafyllou T., Michalinos A., Kamberoglou D., Tsekrekos A., Rouvelas I. (2020). Pseudoachalasia: A systematic review of the literature. Esophagus.

[3] Gockel H.R., Schumacher J., Gockel I., Lang H., Haaf T., Nöthen M.M. (2010). Achalasia: Will genetic studies provide insights?. Hum. Genet..

[4] Furuzawa-Carballeda J., Torres-Landa S., Valdovinos M.Á., Coss-Adame E., Martín Del Campo L.A., Torres-Villalobos G. (2016). New insights into the pathophysiology of achalasia and implications for future treatment. World J. Gastroenterol..

[5] Grover S., Gockel I., Latiano A., Mokrowiecka A., Dasmeh P., Wouters M.M., Vackova Z., Haas S.L., Triantafyllou T., Kreuser N. (2025). First genome-wide association study reveals immune-mediated aetiopathology in idiopathic achalasia. Gut.

[6] Di Bella D., Lazzaro F., Brusco A., Plumari M., Battaglia G., Pastore A., Finardi A., Cagnoli C., Tempia F., Frontali M. (2010). Mutations in the mitochondrial protease gene *AFG3L2* cause dominant hereditary ataxia SCA28. Nat. Genet..

[7] Galatolo D., De Michele G., Silvestri G., Leuzzi V., Casali C., Musumeci O., Antenora A., Astrea G., Barghigiani M., Battini R. (2021). NGS in Hereditary Ataxia: When Rare Becomes Frequent. Int. J. Mol. Sci..

[8] Estiar M.A., Yu E., Haj Salem I., Ross J.P., Mufti K., Akçimen F., Leveille E., Spiegelman D., Ruskey J.A., Asayesh F. (2021). Evidence for Non-Mendelian Inheritance in Spastic Paraplegia 7. Mov. Disord..

[9] Müller U. (2021). Spinocerebellar ataxias (SCAs) caused by common mutations. Neurogenetics.

[10] Cohen B.H., Naviaux R.K. (2010). The clinical diagnosis of POLG disease and other mitochondrial DNA depletion disorders. Methods.

[11] Béreau M., Anheim M., Echaniz-Laguna A., Magot A., Verny C., Goideau-Sevrain M., Barth M., Amati-Bonneau P., Allouche S., Ayrignac X. (2016). The wide POLG-related spectrum: An integrated view. J. Neurol. Sci..

[12] Weiss M.D., Saneto R.P. (2010). Sensory ataxic neuropathy with dysarthria and ophthalmoparesis (SANDO) in late life due to compound heterozygous POLG mutations. Muscle Nerve.

[13] Drokhlyansky E., Smillie C.S., Van Wittenberghe N., Ericsson M., Griffin G.K., Eraslan G., Dionne D., Cuoco M.S., Goder-Reiser M.N., Sharova T. (2020). The Human and Mouse Enteric Nervous System at Single-Cell Resolution. Cell.

[14] Boeckxstaens G.E. (2008). Achalasia: Virus-induced euthanasia of neurons?. Am. J. Gastroenterol..

[15] Hallal C., Kieling C.O., Nunes D.L., Ferreira C.T., Peterson G., Barros S.G., Arruda C.A., Fraga J.C., Goldani H.A. (2012). Diagnosis, misdiagnosis, and associated diseases of achalasia in children and adolescents: A twelve-year single center experience. Pediatr. Surg. Int..

[16] Camilleri M. (2021). Gastrointestinal motility disorders in neurologic disease. J. Clin. Investig..

[17] Chen W., Zhao H., Li Y. (2023). Mitochondrial dynamics in health and disease: Mechanisms and potential targets. Signal Transduct. Target. Ther..

[18] Somai S., Aloh C.H., King D.E., Copeland W.C. (2025). Mitochondrial DNA replication and disease: A historical perspective on molecular insights and therapeutic advances. Int. J. Mol. Sci..

[19] Rahman S., Copeland W.C. (2019). POLG-related disorders and their neurological manifestations. Nat. Rev. Neurol..

[20] Finsterer J., Frank M. (2017). Gastrointestinal manifestations of mitochondrial disorders: A systematic review. Ther. Adv. Gastroenterol..

[21] Chelimsky G., Shanske S., Hirano M., Zinn A.B., Cohen M., McNeeley K., Chelimsky T.C. (2005). Achalasia as the harbinger of a novel mitochondrial disorder in childhood. J. Pediatr. Gastroenterol. Nutr..

[22] Kornblum C., Broicher R., Walther E., Seibel P., Reichmann H., Klockgether T., Herberhold C., Schröder R. (2001). Cricopharyngeal achalasia is a common cause of dysphagia in patients with mtDNA deletions. Neurology.

[23] Koppen M., Metodiev M.D., Casari G., Rugarli E.I., Langer T. (2007). Variable and tissue-specific subunit composition of mitochondrial m-AAA protease complexes linked to hereditary spastic paraplegia. Mol. Cell. Biol..

[24] Löbbe A.M., Kang J.S., Hilker R., Hackstein H., Müller U., Nolte D. (2014). A novel missense mutation in *AFG3L2* associated with late onset and slow progression of spinocerebellar ataxia type 28. J. Mol. Neurosci..

[25] Zühlke C., Mikat B., Timmann D., Wieczorek D., Gillessen-Kaesbach G., Bürk K. (2015). Spinocerebellar ataxia 28: A novel *AFG3L2* mutation in a German family with young onset, slow progression and saccadic slowing. Cerebellum Ataxias.

[26] Szpisjak L., Nemeth V.L., Szepfalusi N., Zadori D., Maroti Z., Kalmar T., Vecsei L., Klivenyi P. (2017). Neurocognitive Characterization of an SCA28 Family Caused by a Novel *AFG3L2* Gene Mutation. Cerebellum.

[27] Graziewicz M.A., Longley M.J., Copeland W.C. (2006). DNA polymerase gamma in mitochondrial DNA replication and repair. Chem. Rev..

[28] Filosto M., Mancuso M., Nishigaki Y., Pancrudo J., Harati Y., Gooch C., Mankodi A., Bayne L., Bonilla E., Shanske S. (2003). Clinical and genetic heterogeneity in progressive external ophthalmoplegia due to mutations in polymerase gamma. Arch. Neurol..

[29] Vogel A.P., Rommel N., Oettinger A., Horger M., Krumm P., Kraus E.M., Schöls L., Synofzik M. (2017). Speech and swallowing abnormalities in adults with POLG associated ataxia (POLG-A). Mitochondrion.

[30] Da Pozzo P., Cardaioli E., Rubegni A., Gallus G.N., Malandrini A., Rufa A., Battisti C., Carluccio M.A., Rocchi R., Giannini F. (2017). Novel POLG mutations and variable clinical phenotypes in 13 Italian patients. Neurol. Sci..

[31] Valent A., Delorme L., Roland E., Lambe C., Sarnacki S., Cattan P., Plaud B. (2022). Peri-operative management of an adult with POLG-related mitochondrial disease. Anaesth. Rep..

[32] Costa M., Spencer N.J., Brookes S.J.H. (2021). The role of enteric inhibitory neurons in intestinal motility. Auton. Neurosci..

[33] Jamka J.R., Gulbransen B.D. (2025). Mechanisms of enteric neuropathy in diverse contexts of gastrointestinal dysfunction. Neurogastroenterol. Motil..

[34] Kural I., Mombeek L.M.M., Wilson D.M. (2026). Role of mitochondria in neuronal function and survival in the enteric and central nervous systems. Cell. Mol. Life Sci..

[35] Richards S., Aziz N., Bale S., Bick D., Das S., Gastier-Foster J., Grody W.W., Hegde M., Lyon E., Spector E. (2015). ACMG Laboratory Quality Assurance Committee. Standards and guidelines for the interpretation of sequence variants: A joint consensus recommendation of the American College of Medical Genetics and Genomics and the Association for Molecular Pathology. Genet. Med..

