# Supplementary material for: Rare AFG3L2 and POLG Variants Suggest a Role for Mitochondrial Dysfunction in Enteric Neuronal Vulnerability in Idiopathic Achalasia
Source: Int J Mol Sci. 2026 Jul 28;27(15):6756. doi: 10.3390/ijms27156756 (PMC13466295; doi:10.3390/ijms27156756)
Supplement: Supplementary file 1 [file ijms-27-06756-s001.zip › ijms-4411803-supplementary.pdf]

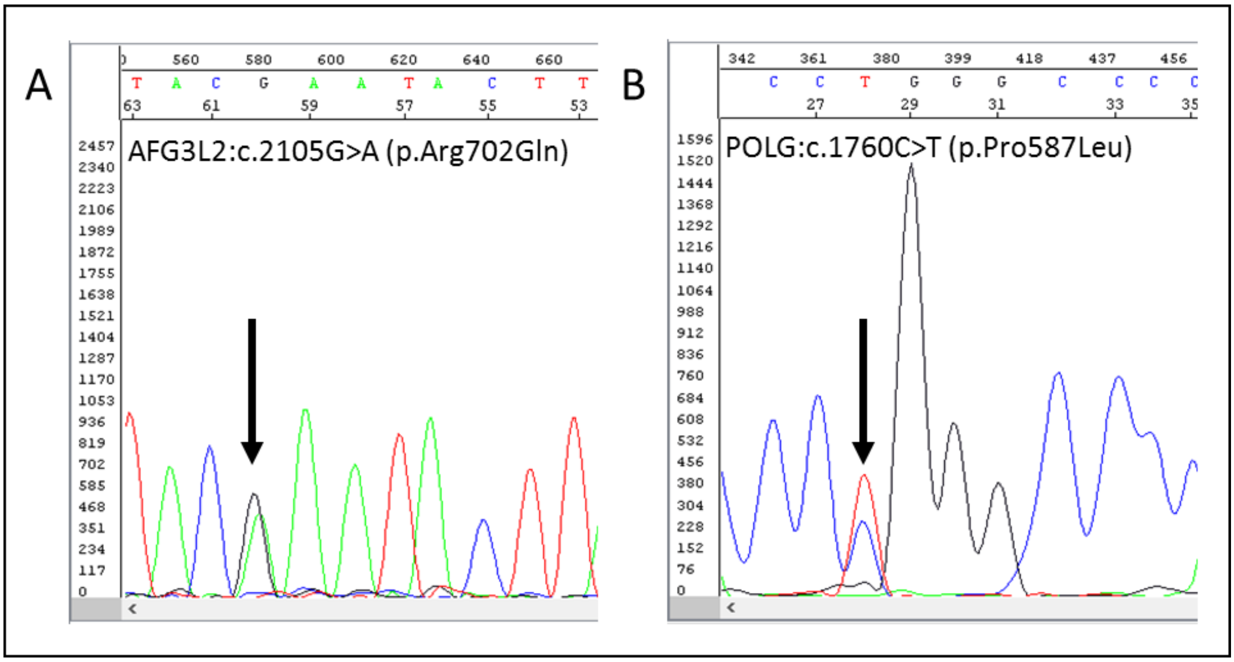

Supplementary Figure S1. Electropherograms of the identified variants. **A.** Patient 1. Heterozygous c.2105G>A variant in exon 16 of *AFG3L2* gene (arrow). **B.** Patient 2. Heterozygous c.1760C>T variant in exon 10 of *POLG* gene (arrow).
